# A test of positive suggestions about side effects as a way of enhancing the analgesic response to NSAIDs

**DOI:** 10.1371/journal.pone.0209851

**Published:** 2019-01-03

**Authors:** Aurore Fernandez, Irving Kirsch, Louis Noël, Pierre Yves Rodondi, Ted J. Kaptchuk, Marc R. Suter, Isabelle Décosterd, Chantal Berna

**Affiliations:** 1 Pain Center, Department of Anesthesiology, Lausanne University Hospital (CHUV), Lausanne, Switzerland; 2 Faculty of Biology and Medicine (FBM), University of Lausanne (UNIL), Lausanne, Switzerland; 3 Program in Placebo Studies, Harvard Medical School, Boston, MA, United States of America; 4 Institute of Social and Preventive Medicine (IUMSP), Lausanne University Hospital (CHUV), Lausanne, Switzerland; La Sapienza University of Rome, ITALY

## Abstract

Side effects are frequent in pharmacological pain management, potentially preceding analgesia and limiting drug tolerability. Discussing side effects is part of informed consent, yet can favor nocebo effects. This study aimed to test whether a positive suggestion regarding side effects, which could act as reminders of the medication having been absorbed, might favor analgesia in a clinical interaction model. Sixty-six healthy males participated in a study “to validate pupillometry as an objective measure of analgesia”. Participants were unknowingly randomized double-blind to positive vs control information about side effects embedded in a video regarding the study drugs. Sequences of moderately painful heat stimuli applied before and after treatment with diclofenac and atropine served to evaluate analgesia. Atropine was deceptively presented as a co-analgesic, but used to induce side effects. Adverse events (AE) were collected with the General Assessment of Side Effects (GASE) questionnaire prior to the second induced pain sequence. Debriefing fully informed participants regarding the purpose of the study and showed them the two videos.The combination of medication led to significant analgesia, without a between-group difference. Positive information about side effects increased the attribution of AE to the treatment compared to the control information. The total GASE score was correlated with analgesia, i.e., the more AEs reported, the stronger the analgesia. Interestingly, there was a significant between-groups difference on this correlation: the GASE score and analgesia correlated only in the positive information group. This provides evidence for a selective link between AEs and pain relief in the group who received the suggestion that AEs could be taken as a sign “that help was on the way”. During debriefing, 65% of participants said they would prefer to receive the positive message in a clinical context. Although the present results cannot be translated immediately to clinical pain conditions, they do indicate the importance of testing this type of modulation in a clinical context.

## Introduction

Commonly used medications in pain management frequently have side effects, *i*.*e*. drug effects that are secondary to the one intended. Side effects often limit drug tolerability or prevent proper introduction especially in the context of chronic pain [1] [2]. In a study of pregabalin for chronic neuropathic pain, 66% of patients (N = 224/338) experienced at least one side effect, and 18% discontinued treatment due to this [3]. Crucially, communication about side effects is delicate: providers have a legal and moral obligation to inform patients fully about their treatment, but they should avoid inducing unnecessary worry or nocebo effects [4, 5]. This necessary discussion could perhaps be used to maximize the benefits of pharmacological treatment or the tolerability of possible side effects ([6]).

Placebo effects are the enhancement of an actual or sham medication’s pharmacological effects through positive expectancy and non-specific factors [7]. Placebo analgesia has been shown to be modulated, for example, by prior treatment experience [8], anxiety [9], somatic focus [10], as well as by information regarding the medication, such as drug value and brand [11–13]. Furthermore, awareness that the therapy is being given impacts clinical effects, as illustrated by open/hidden paradigms where analgesic doses are much less effective if the subject is not conscious of their delivery [14–16]. Evolutionary psychology theories have proposed that self-healing processes could be favored by a third-party permission to recover, such as a ritual or a healer [17]. Here we wondered whether patients could interpret side effects (i.e. noticeable bodily sensations associated to the drug intake) as a third-party signal reminding them that the medication is active in the body, hence conveying a message that “help is on the way”.

There is some evidence to support this hypothesis. A double-blind randomized controlled trial (RCT) of single doses of different agents for post herpetic neuralgia reported a correlation between the number of side effects and pain relief [18]. In a more recent meta-analysis of RCTs for irritable bowel syndrome, the difference in pain between drug and placebo groups correlated with the difference in side effects [19]. The authors hypothesized that “patients feeling the effects of the drug may associate this sensation with that of efficacy” [19]. This hypothesis has been tested in an experimental double-blind RCT of a nonsteroidal anti-inflammatory drug (NSAID) with and without a side-effect induced by atropine, deceptively presented as a co-analgesic [20]. This study was the first experimental demonstration that the perception of side effects in an analgesic RCT affects beliefs about treatment assignment, which in turn increases the response to the medication.

The impact of side effects on therapeutic efficacy may be linked to the instruction that one might receive a placebo. It is unknown if these findings could translate to a therapeutic setting, where the patient is certain to receive active medication. To answer this question, we compared the analgesia provided by an NSAID following a standard information about side effects and an information containing a suggestion that side effects could be considered as a signal of biological assimilation of the compound (i.e. a message of “help is on the way!”). The information was provided in an experimental model of the clinical introduction of an analgesic compound, with side-effect induction through atropine.

## Method

### 1. Ethics statement

The protocol was approved by the local Ethic Committee: Swiss Cantonal Commission for clinical research ethics, Canton Vaud, Switzerland (study registration number: CER_VD 215/15) and was in accordance with principles of the Declaration of Helsinki, the Essentials of Good Epidemiological Practice issued by Public Health, and the Swiss Law as applicable. All participants gave written informed consent.

### 2. Participants

Participants were recruited via advertisements posted on the University of Lausanne’s website and campus advertisement boards. Men between 18 and 40 years old were phone-screened to be healthy and fluent French speakers. Exclusion criteria were chronic intake of medication, chronic pain, a psychiatric condition, or any condition (e.g. gastritis, high blood pressure, and allergy) with increased risk for NSAIDs or atropine intake. We calculated a-priori that 26 participants experiencing adverse event (AE) per condition would be sufficient to detect an effect size comparable to that obtained in a previous study [d = 0.7], with 80% power [20]. Based on this prior study reporting a 20% AE induction failure of atropine, a target of 33 participants in each group was set. Seventeen screened volunteers were not eligible. The recruited sample consisted of 66 healthy males aged 18–38 years (*M = 24*.*3*, *SD = 4*.*1*). The study was limited to males considering prior conflicting literature regarding differential gender-based responses to NSAIDs and placebo modulations [21] [22]. Participants received CHF 50 to compensate for their time.

### 3. General procedure

The study was presented as aiming to “evaluate the subjective relief brought by a drug combination, and the correlation with an objective measure of pain, called pupillometry”. The pupillometer (Neurolight Algiscan, IDMed, France) is a device validated for assessing reactivity to noxious stimuli during anesthesia [23–25]. There is preliminary evidence that pupillometry can be used as an objective measure of intense nociceptive stimulation in conscious people [26, 27]. Little is known about lower intensity stimulation in an experimental context. Hence, pupillometry was used as a cover story and as an exploratory measure. This partial study disclosure, omitting the randomization to two different conditions of information about drug side effects, was maintained until the end of the study procedures when debriefing occurred. Testing took place at the Pain Center at the CHUV, Lausanne, Switzerland, between May and October 2016. Participants were invited for a single session lasting about 2h30 and were asked not to eat any solids in the hour before coming to the Center in order to avoid interference with medication absorption.

**Fig 1:**
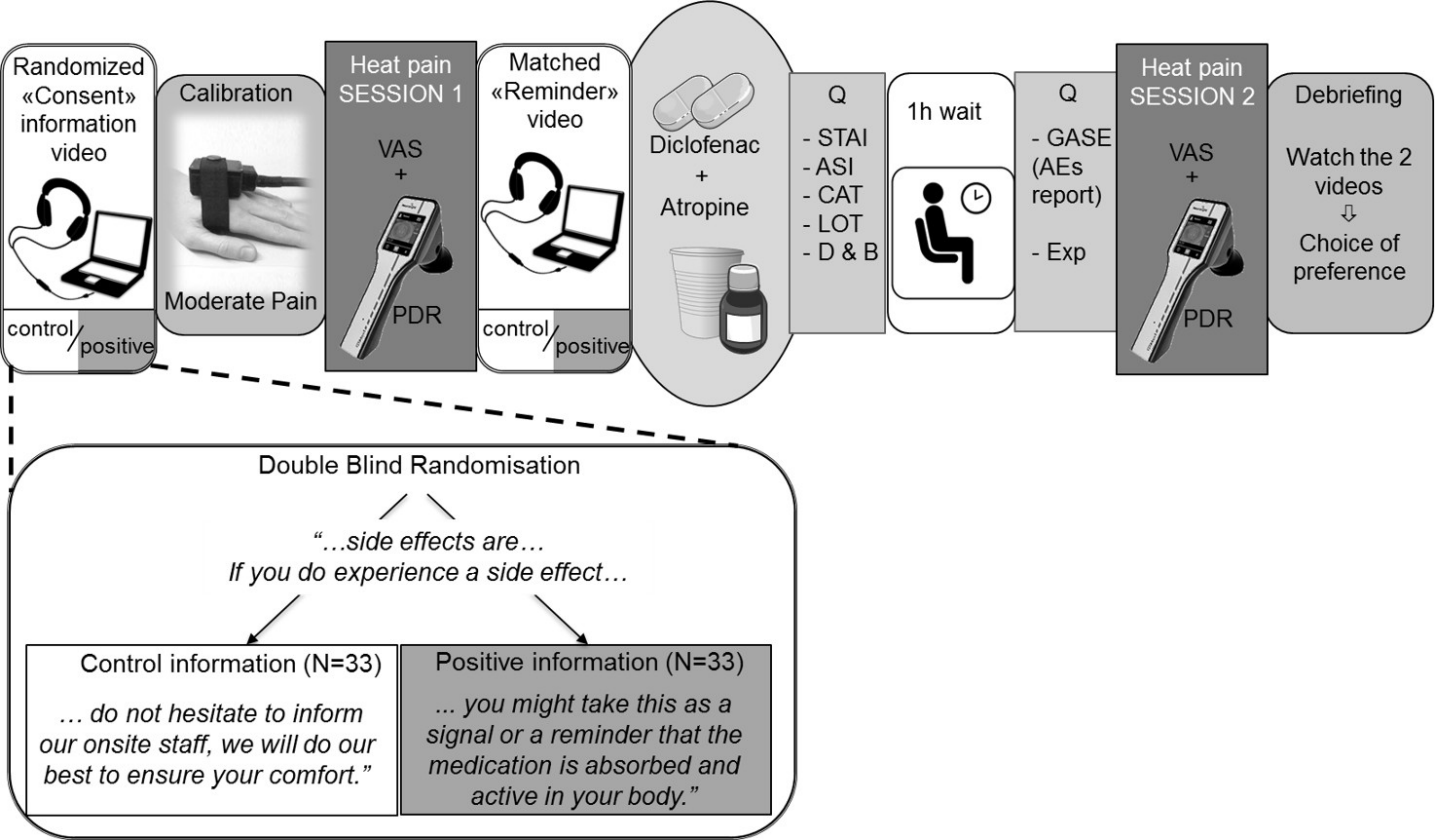
General procedure. Participants were randomized double-blindly to one of the two different information conditions, watching a “consent” information video prior to signing consent. In this video, a physician described the medication’s effects and side effects. The only difference between the two messages concerned side effects and is summarized in the verbatim. After a calibration to determine the temperature eliciting moderate pain, heat pain session 1 took place, with pain intensity Visual Analogue Scales (VAS), and pupillary dilation reflex (PDR) measures. Then, the “reminder” video regarding side effects was watched before taking the study medication. At the beginning and end of the 1-hour long break (while the medication was becoming active), participants filled Questionnaires (Q). A second heat pain testing ensued, and the study concluded with a debriefing. STAI = state-trait anxiety inventory, ASI = anxiety sensitivity Index, CAT = catastrophizing, LOT = life orientation test, D&B = desire and beliefs about medication, GASE = General Assessment of Side Effects, Exp = expectation.

Unbeknown to them, participants were randomized 1:1 to positive vs. control information about the experience of side effects provided through pre-recorded video messages (see details of the information modulation below). Investigators involved in recruitment and testing (A.F., L.N., C.B.) were blind to the information condition. A collaborator (M.S.) prepared a coded, randomized sequence of video assignments. The videos were shown on a laptop with a noise canceling headset (see Fig 1).

First, vital signs were assessed and eligibility criteria were confirmed. Then the procedure was explained to ascertain understanding; the first 3 min-long video was shown, and informed consent was taken. In the video, the risks and benefits of the medications were disclosed. However, atropine was presented deceptively as an analgesic, whereas it was actually used to induce AEs, mostly dry mouth [20]. Participants underwent heat pain calibration and a first test run of experimental heat pain, used as a baseline. After watching a 1-minute long video-reminder, all the participants received atropine 1.2mg (Streuli Pharma, Uznach, CH) and diclofenac 100mg (Novartis Pharma Schweiz AG) taken orally (Fig 1). Participants then went to a waiting room where they filled questionnaires for 15 minutes (hope for relief, beliefs about medication potency, anxiety, optimism, catastrophizing, anxiety sensitivity), followed by a 45 min wait for the medication to fully become active. Salivary flow significantly decreases 50 min after administration of atropine [28], peak anti-inflammatory activity occurs 60 min after administration of diclofenac [29]. After these 60 min, back in the testing room, participants reported AEs on a standardized questionnaire [30] as well as a measure of expectation. Then they proceeded with the post-treatment experimental heat pain testing. Finally, participants were fully debriefed regarding the additional purpose of the study and the effects of atropine.

### 4. Information modulation

Two sets of video-recorded messages (the main “consent” information video and the shorter pre-drug intake “reminder”, see Fig 1) provided information about the study medications in French by a 41-year-old male physician. The first 3-min video contained information about the combination of diclofenac and atropine: mechanism of action (deceptive regarding the atropine, presented as a co-analgesic), administration, and side effects. The second 1-min video described the medication administration, pharmacokinetics, and a reminder about the side effects. There were two versions of each of these videos, one containing a positive message about side effects and a control version without this positive message.

The positive and control video sets were identical except for one sentence in the segment about side effects. Both started with: “Side effects of this medication combination in healthy volunteers are frequent, usually mild and without consequences. The most frequently reported one is dryness of the mouth. In addition, some people experience visual accommodation issues, feeling hot, having a hot, red or dry skin, jitteriness, dizziness or slight concentration disruption. Decreases or increases in heart rate can be observed. All these will resolve within 2–4 hours of taking the medication.” Following this, in the “positive” information condition, participants were told: “If you do experience a side effect, you might take this as a reminder that the analgesic medication is active in your body, and that it will help you to perceive less pain. Perhaps you can consider this as a signal that the drug is working”. In the “control” condition the matching segment was: “If you do experience a side effect, do not hesitate to inform our onsite staff, we will do our best to ensure your comfort". The information ended in both conditions with thanks for participating and an invitation to ask questions to the onsite study team.

### 5. Measures

#### 5.1 Pain stimulation

Initial heat pain stimulus calibration allowed to identify the individual temperature needed to elicit moderate (Mod) pain ratings, i.e. rated about 5 on a 10 cm Visual Analogue Scale (VAS) (Mean VAS rating = 5.1, SD = 1.0). This was performed by applying different test temperatures (ladder method) to a 10-second thermal stimulus delivered on the left forearm (TSA-II, Medoc, Ramat Yishai, Israel) (19, 30) in order to observe a reproducible pain rating. This calibration also ascertained that Mod +1°C was intensely painful but tolerable (Mean VAS rating = 6.5, SD = 1.2), and Mod -0.5°C as well as -1°C were perceived on average, as low pain (Mean VAS rating = 2.7, SD = 1.6).The pre- and post-treatment testing consisted in the same sequence of ten stimuli: **Mod** / **Mod** / Mod-0.5°C / **Mod** / Mod+1°C / **Mod** / **Mod** / Mod-1°C / **Mod** / **Mod**.

Subjects rated the pain intensity of each stimulus on a 10 cm-VAS (with anchors of “no pain” and “most intense pain imaginable”) [31] during 10-30s variable interstimulus intervals.

The mean rating of pain intensity from the 7 repetitions of moderate stimuli (***Mod***) was the outcome of interest, with the other 3 interspersed stimuli intended as distracters [20, 32]. Analgesia was calculated as the difference in the mean of the 7 VAS (Mod) between pre- and post-treatment sequences.

#### 5.2. Adverse events reporting

One hour after medication intake, the participants filled the Generic Assessment of Side Effects (GASE) [30]. This standardized questionnaire requires the participant to rate the severity of 36 adverse events (AEs) on a scale (0 = not present, 1 = mild, 2 = moderate, 3 = severe) and to categorize each present AE as related to the study medication (attributed AE) or not. This allows computing four indices: 1) A count of reported AE: with a score ≥1 (Number of AE), 2) number of AE attributed to the medication (attributed AE), 3) total GASE score (sum of intensity ratings on all items), and 4) Medication-attributed GASE score (Rief, Glombiewski & Barsky 2009: Generic Assessment of Side Effects. www.GASE-scale.com). A ‘mean intensity of AEs’ was computed by dividing the GASE by the number of AEs, considering only participants with ≥1 AE, both for total and attributed AEs. As AEs were not expected to be severe, but could be unpleasant, an additional measure of AE unpleasantness was collected on a VAS, with anchors from 0 (not at all) to 10 (intolerable).

#### 5.3. Pupillometry

The pupillary reflex was measured by an infrared portable dynamic pupillometer (NeuroLight). Measurements were performed with the device’s rubber cup covering the measured eye (left eye) and the participant’s right hand covering the right eye. The device assesses the baseline pupillary size as well as the pupillary dilatation reflex (PDR), *i*.*e*. the variation of the pupillary diameter over the course of the painful stimulation, expressed in percent. The recording was initiated as the thermal stimulus started, and stopped after 12 seconds. This measure was repeated 5 times using a second, shorter sequence of stimuli (**Mod** / **Mod** / Mod-0.5°C / **Mod** / Mod+1°C). This sequence of measures occurred right after the pre- and post-treatment sequence of 10 stimuli with the VAS ratings. Only pre-treatment PDR measures were analyzed as atropine can affect the post-treatment pupillary diameter and PDR.

#### 5.4. Desire, beliefs, expectations, and psychological characteristics

An effect of the information modulation on desire for relief beliefs, and expectations was measured [33, 34]. Participants rated: 1) just after taking the medication “How much would you like to be relieved by the combination of medication?” as well as: “How efficient do you believe the medication combination to be against heat pain?” on 10-cm VAS anchored from “not at all” to “extremely” and 2) after having reported AEs, “How much relief do you expect from the combination of medication?” on a VAS ranging from “no relief” to “complete relief”.

In order to control for possible moderators, anxiety (measured using State and Trait anxiety inventory, STAI (Spielberger, Gorsuch, Lushene, Vagg, & Jacobs, 1983) and Anxiety Sensitivity Index, ASI [35]), optimism (LOT [36]), and in vivo catastrophizing (6-item scale derived from the Pain Catastrophizing Scale [37]) were evaluated. In addition, prior to the first calibration, participants gave a baseline VAS evaluation of their anxiety, focus, and tiredness. All data were collected and managed using REDCap electronic data capture tools hosted at the University of Lausanne [38].

#### 5.5. Debriefing preference regarding information and estimation of the effects of information

During the final debriefing, participants were shown the two different “reminder” video segments about side effects. Afterward, they were asked which type of information (positive or control) they would prefer to receive from their doctor at the beginning of a new treatment. They were asked not to disclose which one they saw during the test sequence.

Finally, participants’ estimation regarding a potential effect of the positive information on the efficacy of the treatment “Do you think that this explanation could enhance the effects of the treatment?” was also rated on a VAS ranging from 0 (no effect at all) to 10 (very much effect).

### 6. Statistical analysis

The primary outcome was the reduction in moderate pain intensity following treatment (i.e. analgesia, post- minus pre-treatment moderate pain ratings). We used a 2x2 (group by treatment) mixed model ANOVA to test for differences in pain scores with group as a between subject factor and pre- versus post-treatment as a repeated measure. The secondary outcomes were AEs reports (unpaired t-tests for group comparison) and the relationship between analgesia and AEs (Pearson’s correlations). The Group factor was examined as a potential moderator of the correlation between AEs and analgesia, by regressing analgesia on the interaction term (group x AEs), controlling for group and number of AEs.

In order to assess possible baseline group differences despite randomization, participant’s characteristics were compared with unpaired t tests.

As a further manipulation check, chi-square tests of homogeneity were calculated comparing the preference of information between groups.

Exploratory analyses were conducted without correction for multiple comparisons except for the pupillometry data: a) The relationships between analgesia and measures of desire, expectations, and beliefs (Pearson’s correlations). b) The relationship between psychological variables (anxiety, optimism, catastrophizing) and the number of AEs was explored through Pearson’s correlations. c) VAS and PDR between low, moderate, and intense stimuli were compared through ANOVAs followed by Bonferroni corrected post-hoc multiple comparisons and correlated using Pearson’s correlations.

SPSS 23.0 (IBM Statistics) was used to conduct these analyses.

## Results

### 1. Participant characteristics

The participants (N = 33 healthy males in each group) randomized to the different information conditions (positive or control) did not differ significantly in age, temperature eliciting moderate pain, pre-treatment moderate pain rating, optimism, anxiety, catastrophizing, beliefs about the medication potency, desire for, or expectations of relief *(See Table 1).*

**Table 1:** Participants’ characteristics.

	Control group *(n = 33) M (SD)*	Positive group *(n = 33) M (SD)*	Group difference p value	Correlation with analgesia r; p value
Age, *years*	24.2 (4.0)	24.5 (4.3)	.76	-.12; p = .33
Anxiety, *NRS/10*	1.7 (2.1)	2.4 (2.4)	.25	.06; p = .64
Focused, *NRS/10*	7.7 (1.7)	7.6 (1. 8)	.78	-.00; p = .98
Tired, *NRS/10*	3.8 (2.1)	3.2 (2.1)	.27	-.02; p = .87
Calibration for moderate pain, °C	46.4 (1.7)	46.0 (1.6)	.33	-.04; p = .76
Pre-treatment pain ratings, *VAS/10*	4,8 (1.2)	5.0 (0.8)	.47	-.14; p = .25
State-Trait Anxiety Inventory-State *(STAI-S)*	28.3 (8.8)	30.9 (8.6)	.22	.06; p = .61
State-Trait Anxiety Inventory-Trait *(STAI-T)*	34.2 (9.0)	37.0 (9.2)	.20	-.03; p = .82
Anxiety Sensitivity Index *(ASI)*	15.3 (7.5)	18.2 (8.0)	.14	.12; p = .32
Pain catastrophizing scale *(CAT)*	1.6 (1.7)	2.3 (2.3)	.19	.09; p = .47
Life Orientation Test *(LOT)*	16.6 (4.8)	15.1 (4.2)	.17	.06; p = .64
Desire for relief (VAS 0–10)	6.4 (2.2)	6.7 (2.3)	.61	.18; p = .14
Belief of efficacy (VAS 0–10)	6.0 (1.6)	6.0 (1.9)	.95	.05; p = .69
Expectations of relief (VAS 0–10)	5.9 (1.9)	6.5 (4.1)	.42	.03; p = .81

M = mean, NRS = numerical rating scale; VAS = visual analogue scale.

### 2. Analgesia

**Fig 2:**
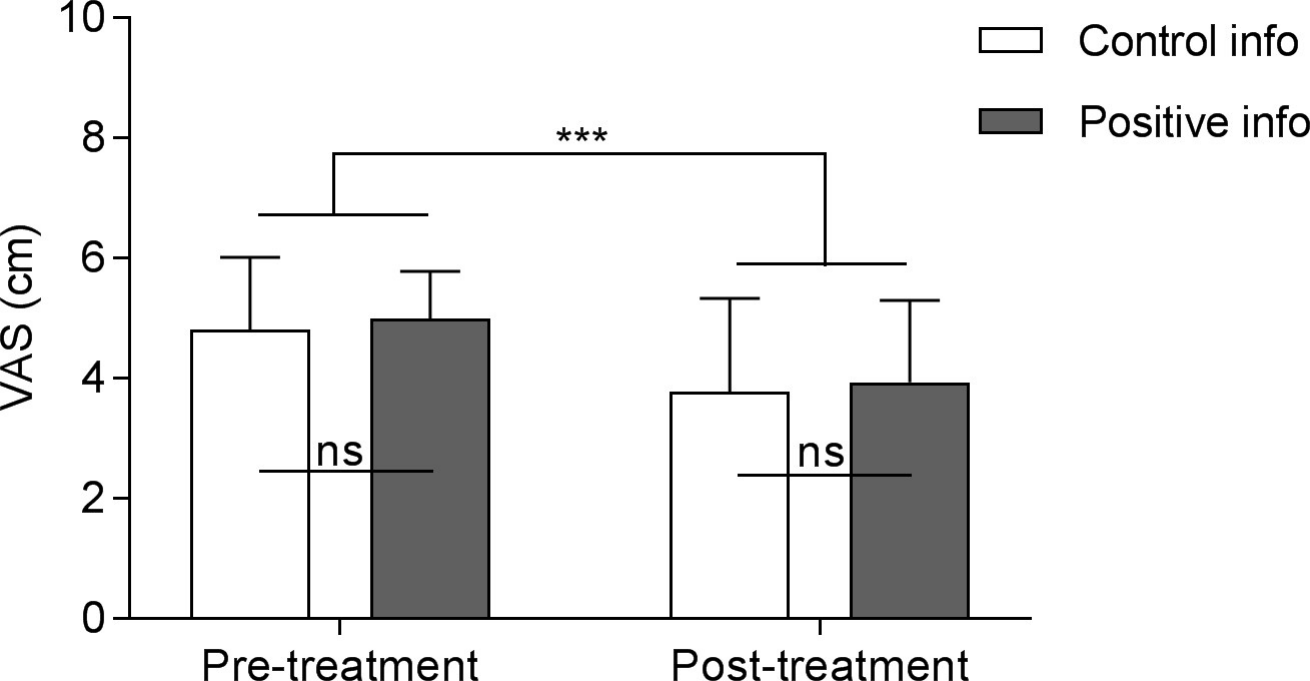
Pain intensity scores before and after treatment, according to information group. The analgesic and atropine combined treatment had a significant effect on pain intensity ratings of moderate heat pain stimuli (mean of seven stimuli) without significant difference between information groups regarding adverse events (either positive or control information). VAS = Visual Analogue Scale; Error bars are SD; *** p< .0001.

Treatment induced analgesia in both positive (M = -1.05, SD = 1.22) and control (M = -1.03, SD = 1.17) information groups (i.e. a significant effect of the treatment) [F(1,32) = 48.76, p < .001], without a significant group effect [F(1,32) = .34, p = .57] or group by treatment interaction [F(1,32) = .01, p = .93] (Fig 2). The same analysis led in the population of participants who did experience adverse events (AEs) presented similar results (significant treatment effect: [F(1,27) = 53.92, p < .001]; no group effect: [F(1,27) = .00, p = .99]; nor interaction: [F(1,27) = .00, p = .99]).

### 3. Adverse events

**Fig 3:**
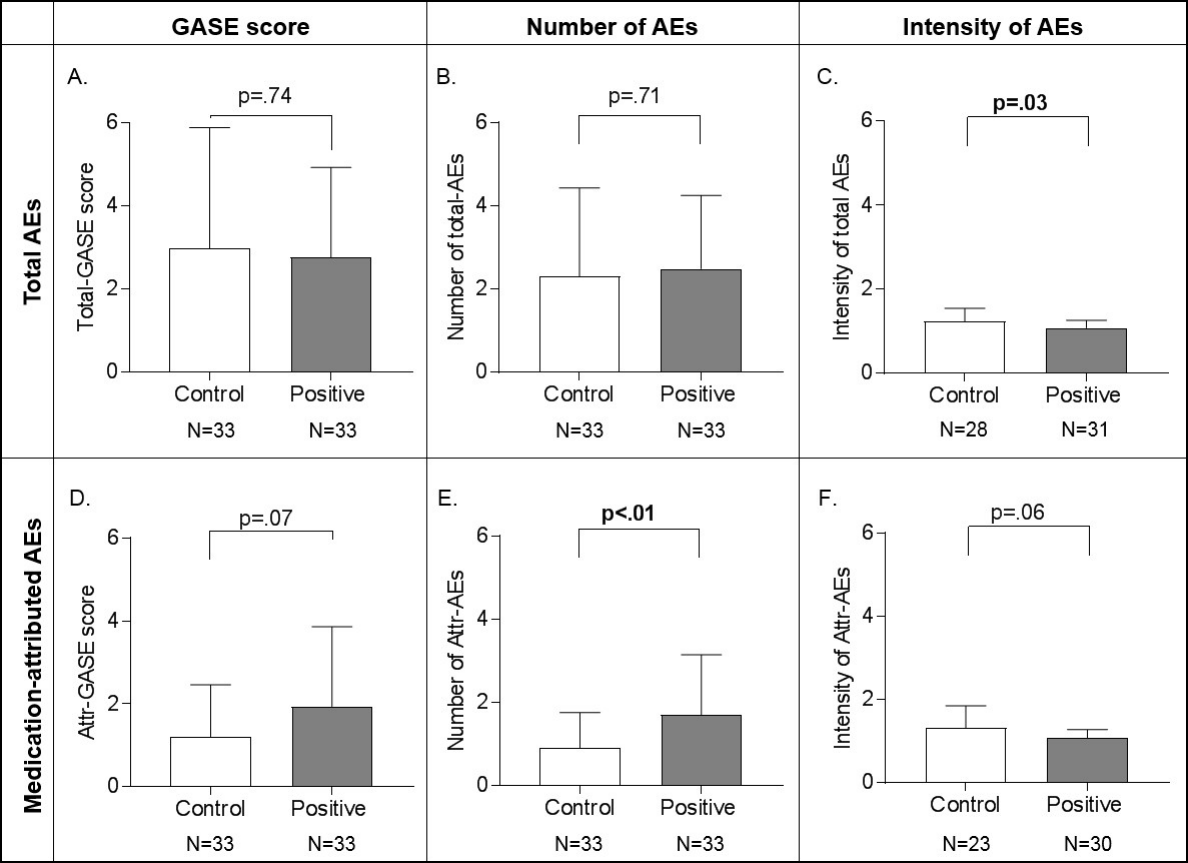
Group comparisons of the Generic Assessment of Side Effects (GASE) score, and its sub-components (number of adverse events (AEs) and intensity) for total and attributed AEs. For total AEs, the GASE score and number of AEs was not significantly different between information groups (A,B), however, the intensity of AEs was stronger in the control group (C). For the attributed AEs, the GASE score difference was not significant (D), the number of AEs attributed to the treatment was higher in the positive group (E). There was no significant between group difference in the mean intensity of attributed AEs (F). Note that the intensity of AEs (E) (and attributed AEs (F)) is only calculated for those who reported AEs (and attributed AEs to the medication (F)), hence the decreased N of participants. Error bars represent mean +/- SD.

The majority of participants (59/66, i.e. 89%) reported at least one adverse event (AE) 1h after medication intake, the most frequent ones were dry mouth (49/66), fatigue (16/66), headache (10/66), and vertigo (6/66). Between-group differences in adverse events are presented in Fig 3. The total GASE score was not significantly different between the two groups (control: M = 2.97, SD = 2.91 vs positive: M = 2.76, SD = 2.16, t(64) = .34 p = .74). The between-group difference in medication-attributed GASE scores failed to reach statistical significance (control: M = 1.21, SD = 1.24 vs positive M = 1.94, SD = 1.92), t(64) = -1.83 p = .07.

Given that the GASE is a composite score combining the number of side effects and their intensity, these factors were studied separately. There was no significant difference in mean number of total AEs reported by the positive (M = 2.49, SD = 1.77) vs the control group (M = 2.30, SD = 2.13); t(64) = .38 p = .71 but those in the positive information group attributed significantly more AEs to the treatment (M = 1.70 SD = 1.44) than those in the control information group (M = .91 SD = .84); t(64) = -2.70 p < .01 (Fig 3). The mean intensity of the perceived AEs was significantly higher in control group (control: M = 1.23 SD = .32 vs positive M = 1.08 SD = .18), t(42.4) = 2.2 p = .03, but intensity differences in AEs attributed to treatment (control: M = 1.32 SD = .53 vs. positive M = 1.08 SD = .20) was not significant, t(26.6) = 2 p = .06. Hence, the positive information group reported more AEs, with lesser intensity. The mean and maximum unpleasantness of these attributed AEs did not differ significantly between groups (Mean unpleasantness: M_Postive_ = 1.47, SD = 1.29; M_Control_ = 1.63, SD = 1.80, t(63) = .42 p = 0.06; Maximum unpleasantness: M_Postive_ = 1.75, SD = 1.95 vs M_Control_ = 1.77 SD = 1.75, t(63) = -.05 p = .49).

### 4. Interaction between analgesia and adverse events

**Fig 4:**
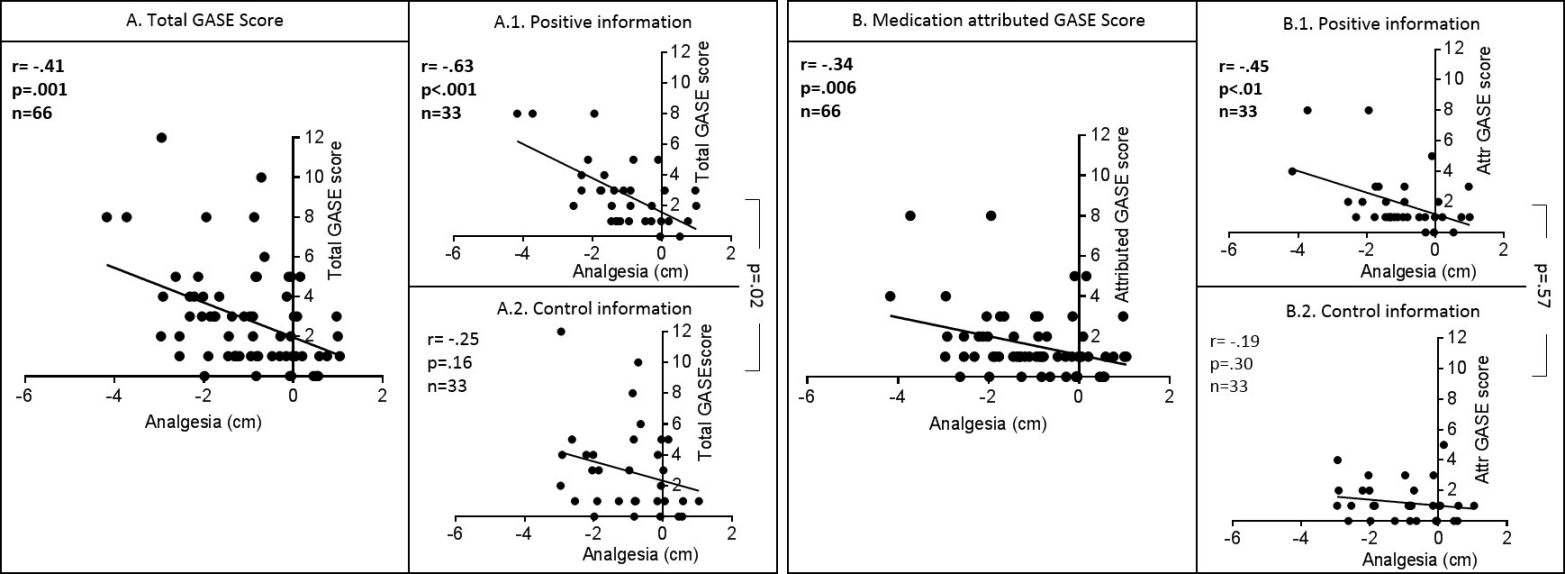
**Correlations between analgesia and (A) the total GASE score and (B) the medication-attributed GASE score.** Significant negative correlations were found between analgesia (VAS post–VAS pre-treatment) and total GASE score, and medication-attributed GASE score, i.e. more analgesia was perceived when more side effects were reported. Yet, this correlation was significant only in the positive information groups (A1, B1). The difference in correlations between the positive and control information group was significant for the correlation between analgesia and the total GASE score, but not for the medication attributed GASE score (comparison A1 vs. A2 and B1 vs. B2).

Both total GASE score and medication attributed GASE score were correlated with analgesia (Fig 4); *i*.*e*., the more AEs are perceived (whether attributed to treatment or not) the stronger the analgesia (total GASE: r = -.41 p = .01; medication-attributed GASE: r = -.34 p < .01). Group differences in correlations were significant for total GASE score (interaction term p = .02), with a correlation between GASE score and analgesia significant in the positive group (r = -.63 p < .001) but not in the control one (r = -.25, p = .16). Differences in correlations were not significant for medication attributed GASE score (interaction term = .57).

### 5. Debriefing questions: Preferred information and estimation of the effect of information

During the debriefing, after watching consecutively the two different abbreviated video segments about side effects, 65% (43/66) of the participants said they would prefer to receive the positive message in a clinical context. Subjects in the positive group were more likely to prefer the positive information than those in the control information group. In the positive group, 85% preferred the positive information whereas in control group 45% preferred the positive information, X^2^(1, N = 66) = 11.28, p = .001.

There was no significant difference between positive and control information groups in participant’s debriefing estimation of potential benefits of the positive information on the efficacy of the treatment, all finding it rather effective (no effect at all = 0, very much effect = 10) [M_positive_ = 6.1 SD = 2.24; M_neutral_ = 5.46 SD = 2.51; t(64) = -1.1, p = .28]. However, there was a correlation between medication-attributed GASE score and participant’s belief of efficiency (r = .31, p = .01) i.e. the more AEs people attribute to the medication, the more effective they think the positive information is at improving treatment outcomes.

### 6. Expectations and measure of desire and beliefs

Measures of expectations, desire and belief did not correlate with analgesia (Table 1), nor with the total GASE score (expectation: r = -.01, p = .92; desire: r = .17, p = .18; beliefs: r = .12, p = .33). These different expectation-related measures collected at different time points correlated with each other (desire X beliefs: r = .49, p < .001; desire X expectation: r = .35, p < .01; beliefs X expectation r = .38, p = .01).

### 7. Psychological variable scores

Correlations indicated that the less optimistic one is (LOT score) the more AEs one perceives (total GASE score; r = -.27; p = .029) and one attributes to medication (medication-attributed GASE score; r = -25; p = .048). Anxiety sensitivity (ASI score) was also correlated with the total GASE score (r = .26; p = .035) and the medication-attributed GASE score (r = .30; p = .016) indicating the more anxious one is, the more side effects one perceives and attributes to the medication. There was no significant correlation between the other psychological scores and AEs reporting.

### 8. Correlation between pre-treatment pupillometry and subjective pain scores

**Fig 5:**
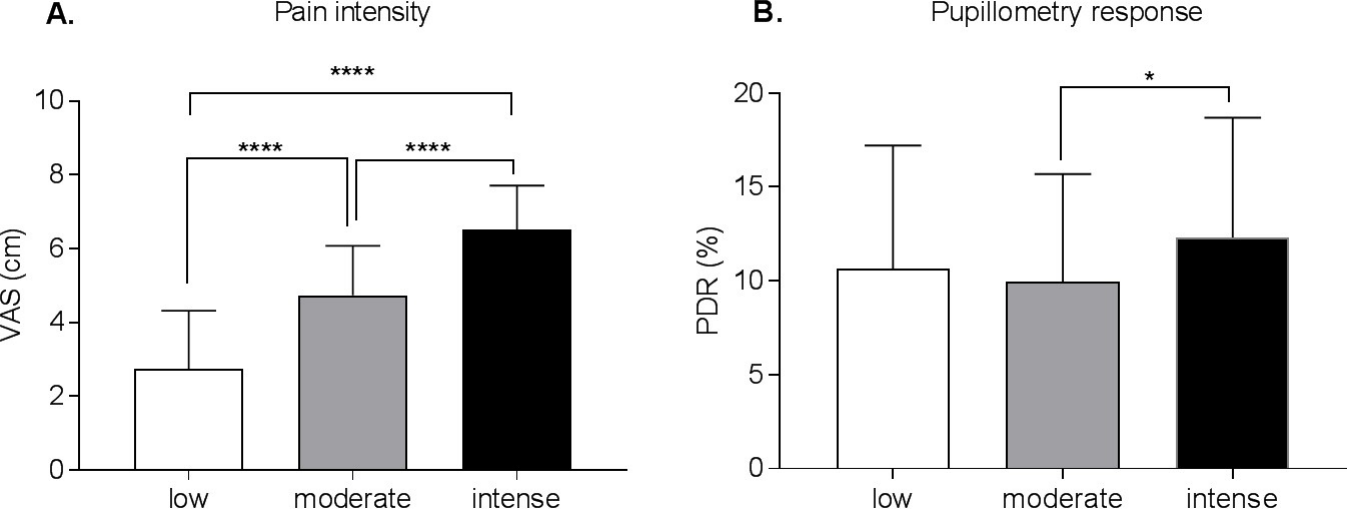
**Pain intensity ratings (panel A) and pupillometry response (panel B) depending on the calibrated stimuli that were applied.** The pain VAS ratings differed significantly between the applied stimuli that were calibrated as low, moderate and intense (A). Only moderate and intense stimuli conditions elicited significantly different pupillometry responses (B). VAS = visual analogue scale; PDR = pupillary dilatation reflex; **** p < .0001 * p < .05.

There were significant effects of intensity on VAS [F (2, 525) = 180.3, p < .0001] and on PDR [F (2, 327) = 3.75, p = .03]. VAS scores were significantly different for low, moderate, and intense conditions (adjusted p<0.001). For the PDR measure, only moderate and intense conditions were significantly different (adjusted p = .02) (Fig 5). There was unsurprisingly no significant correlation between VAS and PDR (low stimuli: r = -.01, p = .94; moderate stimuli: r = -.01, p = .91; intense stimuli:r = -.23,p = .07).

## Discussion

This study aimed at framing side effects as a signal of assimilation of the medication in order to enhance NSAID analgesia. Although the combination of medication led to significant analgesia, the between-group difference was not significant. On the other hand, positive information about side effects did increase the number of them attributed to the medication. Furthermore, a correlation was observed between analgesia and the perceived side effects (GASE score) in the positive information group but not in the control one. This suggests that positive information did specifically tie side effects to analgesia, as hypothesized. After debriefing, and watching both information segments about side effects, a majority preferred the positive one.

Interestingly, the positive framing of side effects led to more attribution of adverse events to medication. Participants reported more numerous AEs compared to the control information suggesting that the positive information about side effects has led to hypervigilance. The quantity and contents of information about side effects can play a critical role in side effect reporting and attribution [4, 39, 40]. In our case, both groups received an equal amount of information. However, we devised a sentence-long positive interpretation of their occurrence, suggesting they act as a reminder that the medication has been ingested, intended to favor a placebo-like effect. This suggestion was rooted in research showing that medical rituals and other signs of active treatment can induce favorable expectations or a positive bias [15, 17, 41]. Also, side effects have been shown to lead to unblinding and to increased analgesia in a prior experimental model of clinical trial [20]. To our knowledge, this is the first study that used verbal suggestions to tie side effects and analgesia in a clinical interaction model. Side effects rely on bodily sensations experienced after the intake of a drug and that are subsequently attributed to it (or not). Side effect attribution is thus an important aspect in their report [42, 43] and is a frequent reason for withdrawing from a clinical trial or discontinuing a medication. The tested information may have led to detection at lower threshold, hypervigilance, or over-attribution of side effects since they were described as a favorable event in our study. Also, negative affect and anxiety have been linked to increased side effect perception and attribution to medication [5]. In fact, our exploratory analysis revealed a correlation between anxiety sensitivity and side effects reporting.

Our aim was to enhance analgesia in the positive information group compared to control group, which we failed to corroborate. Yet, analgesia was higher for those experiencing more side effects, as revealed by the correlation of analgesia with the GASE score in the positive but not in the control information group.

Various reasons could explain why the increased analgesia was not found in the whole positive information group, as initially hypothesized. One could argue the message was too subtle: only one sentence, embedded in a 3-min information video and in a 1 min reminder segment, suggested a positive link between side effects and analgesia. Yet group-differences were found, and perhaps emphasizing the suggestion more would have felt unnatural.

A second possibility is that atropine induced too few and too mild AEs in each participant. In a previous study, atropine led to noticeable symptoms in about 80% of subjects; this was taken into account in the power calculation (see “participants” in methods). Here, as expected, thirteen participants did not experience any AEs, yet with an uneven repartition of this failure to induce AEs: 10 (30%) in the control group, 3 (9%) in the positive group. About half of the participants experienced only dry mouth (34/66), and a small minority (11%) experienced more than two AEs. Given the correlation between number of AEs per participant and experienced analgesia, perhaps this is an important (although unexpected) factor. Possibly, participants with few AEs doubted the positive information, undermining the suggestion. Perhaps inducing stronger or more side effects would have shown group differences in analgesia. Yet this might be practically difficult and ethically questionable. Nevertheless, in a clinical context, side effects are much more relevant. Hence, an intervention focusing on patients at risk of side effects, based on personality traits [5] and tying them to outcomes could possibly be useful.

During debriefing, participants in the positive information group preferred the information suggesting that side effects act as reminders of the medication’s activity in the body, whereas those in the control group did not. Furthermore, when asked to evaluate how much the positive information could enhance the effects of a medication in a hypothetical clinical scenario, all participants gave on average a high rating, often spontaneously mentioning possible placebo effects. Interestingly, in both groups, the more participants perceived side effect, the higher they rated the possible benefits of such information on treatment outcomes. This could have to do with a bias induced by experience (“preferring/justifying the outcome one has received” [44]). Participants who preferred the positive information argued that the message was “reassuring”, “more optimistic”, and “helped to prepare themselves to side effects and to accept them”. Those who preferred the control information mostly argued against the positive message, which “forces the interpretation”, “is a manipulation”, “induces side effects”, and “trivializes side effects”. Some of them hypothesized that “if a patient does not experience side effects, he could think the medication is not active”. In fact, the three participants in the positive information group, who reported no AEs presented almost no analgesia (mean: -0.07 point difference on the VAS score vs. full sample mean: -1.05).

Finally, it may be that an enhancement of therapeutic outcomes by side effects could be limited to RCTs: it has been shown in a prior study that their perception can be used by participants to detect whether they have been randomized to drug or placebo, and thereby benefit from increased analgesia [20]. Yet, we do find a correlation between side effect reports and analgesia that is specific to the group having been exposed to the positive information. This suggests an effect that might require greater power to be detected more widely. In addition, as the sample size was calculated based on the primary outcome (analgesia), without considering the uneven repartition of adverse events, some of the secondary outcomes, such as the correlation analysis might be underpowered. Additional experiments should be performed on larger samples to fully explore the interaction between information, side effects and analgesia, allowing to include measures of psychological traits on the analyses.

Expectations, beliefs about medication, and desire for relief were measured as possible mediators of positive information about side effects on analgesia since treatment effects can be modulated by these factors [33, 45]. However, these elements did not differ between the two groups of information, nor did they correlate with side effects or analgesia, although these exploratory analyses might be underpowered. Hence, we did not manage to capture the mediating factor impacted by the positive information regarding side effects. The relatively low correlation between beliefs about medication potency and expectation of relief is interesting, since they are similar constructs, both of which might be used as a measure of expectancy. This might reflect a change of expectations between measures collected before medication intake (belief and desire) and after noticing side effects (the expectation of relief). However, the difference in wording between the two measures makes it impossible to test directly.

Lastly, this study delivered suggestions through a video-taped message. Previous studies have shown that multimedia communications are well understood and retained, suggesting this is not a limitation to participants’ ability to integrate the provided information [46, 47]. In the debriefing, none of the participants questioned the credibility of the video nor of the physician appearing in it. Nevertheless, pre-recorded communication meant the message was detached from a relationship with a treating physician or a study experimenter which might have implications on the power of the suggestion [48]. In fact, although the physician had the necessary characteristics to build a good relationship, i.e. he appeared empathic, serious and reassuring, [49, 50], he was not present in the room. Whether the practitioner can efficiently incarnate the “heroic rescuer” who can “facilitate a placebo effect” (in the words of [51]) also on a screen, is an open question to be answered by future studies comparing face to face to video-recorded messages.

The video set-up insured proper blinding of the study staff interacting with the participants, contrary to a number of therapeutic suggestion studies where the researcher delivering the message appeared to be aware of the design and research question e.g. [33, 52, 53]. In such prior studies, the unblinded study staff might have enriched the relationship with the participants in the positive condition or deprived those in the control condition. For example, in addition to the scripted suggestion under investigation, empathic touch, and other nonverbal communication could have been modulated [50].

Unsurprisingly, given prior reports of correlations only between very intense painful stimuli and pupillary reflex amplitude, there was no correlation between PDR and VAS ratings [26, 27]. A recent study using an experimental heat pain protocol with healthy volunteers also demonstrated that the pupillary response was more related to the stimulus intensity than to subjective pain perception [54]. As argued by another group, measures of sympathetic autonomic responses might rather reflect nociceptive processes than subjectively perceived pain [55].

In conclusion, thanks to this experimental model, we have answered preliminary and fundamental questions regarding a possible paradigm shift in the communication about side effects. We determined that a positive framing of side effects was credible, even preferred by the majority of participants to whom it is given, and effective in tying them to analgesia, provided there were enough perceived side effects. Although these results are promising, they were obtained in healthy volunteers with transient and mild side effects tied to acute induced pain. Given that these results suggest feasibility and validity of investigating positive communication regarding side effects in a clinical context, the next step could be to evaluate such a framing in actual patients when introducing medication with a high prevalence of side effects.
